# Mr. Wales, on Vaccine Inoculation

**Published:** 1802-06

**Authors:** 


### *Copy of a Letter from* Mr. Wales, *Surgeon of Market Downham, Norfolk.*

To the Editors of the Medical and Physical Journal,

Gentlemen,

At the prefent period it may appear unneceffary to offer to the world farther proofs of the efficacy of Vaccine Inoculation; particularly as the practice has undergone fuch an ample difcuffion in your ufeful publication, fince the period that Dr. Jenner firft made public his facts and obfervations on that fubject. But as there are ftill fome perfons both in and out of the profeffion who affect to entertain fome doubt of its infallibility in preventing the Small-pox, I confider it my duty to fociety to to mention the fuccefs that has uniformly attended my experience, during the application of this mild difeafe to upwards of 540 perfons, refiding at, or within a few miles of this place.

As the fymptoms attending the Cow-pox vary fo little in the different fubjects, and in my practice have not differed from cafes, particularly related in your Journal by other medical gentlemen, it will not be neceffary or ufeful to give more than a general account; fuffice it to fay, I have never feen an inftance, where the patient has been confidered either by myfelf or the family in any danger, nor have I obferved any exceffive pain, ulceration, or any other inconvenience attending the inoculated part, but fuch as have arifen from external injury, by which the puftule has been roughly broken, or from wearing fleeves too tight, and perhaps in female fubjects in particular, made of very harfh materials. With refpect to the perfect fecurity afforded by the Vaccine againft the Variolous difeafe, I can offer the fulleft teftimony, and to the extent of at leaft four out of five of the number before mentioned, having feen that proportion expofed to the latter difeafe, in every poffible way; fome of whom (my youngeft child with the reft) have been inoculated, and others put to bed with perfons in every ftage of the difeafe; whilft a multitude of the lower order of people have been anxioufly intent on bringing the practice into difrepute, and endeavouring, by means the moft filthy* and difgraceful, to produce the Small-pox in thofe who have previoufly gone through the Vaccine under my infpection.

I fhall now proceed to mention a few Cafes in particular, to confirm the facts brought forward by Dr. Jenner, not only as to the fecurity afforded againft the Small-pox, but alfo to prove the length of time which the Cow-pox has been difcovered (in confequence of that gentleman's enlightened and liberal manner of laying his experiments before the public) to have acted in *this county* with the fame influence. I am aware that in bringing forward fome of thefe cafes, I fhall but repeat what have been already before the public in a pamphlet written by Doctor Pearfon, containing "Extract of a letter from me," written two or three years ago; but as they may not be generally known, I fhall here repeat one or two inftances.

——— Rudland, of Weft Dereham, aged 73, (fince dead) never

* An infant, inoculated twelve months before for the Cow-pox, has been made to eat bread and milk after it has been in the mouth of a boy then labouring under a confluent variolous eruption, and very full about the tongue and fauces. I do not mean to infer, that this would have inoculated the infant if not previoufly fecured by the Cow-pox; but that it was expofed *abundantly* to the Small-pox effluvia, without fuftaining the injury intended.

never had the Small-pox; had been a milker all his life; when a boy, remembered to have had a difeafe on his hands and fingers, in confequence of the cows being infected with one which at that time was called the Pap Pox; the cicatrices were evident when I faw him laft, confiderably above fifty years after he had the difeafe: He was not at all aware of any advantages derived from the accident; but when afked, whether he had had the Small-pox, faid No, and fuppofed he never fhould; becaufe he had been inoculated fo many times. He had not feen the diforder in the cow very often, nor at all for fome years, till a month after this converfation, when he fent me word his mafter's cows were difeafed. I went the firft opportunity I had, but the puftules had been all broken by the hands of the milkers, and I did not think proper to take any matter from ulcers fo occafioned.

A very refpectable family in this neighbourhood, originally confifting of three brothers and two fifters, were in the habit of milking more than thirty years ago, contracted a difeafe from the cows, and fuffered fo feverely from numerous puftules on their hands, and great fymptomatic fever from the irritation produced by improper applications to the fores, that they were incapable of any employment, and fent to labourers in the village to milk for them: (one of whom has been before fpoken of as having carried the Vaccine matter to the cornea, by rubbing the eye with a difeafed finger, and having loft his fight from the opacity occafioned by the cicatrix*.) Of this family, two brothers and a fifter are now living within a mile or two of this place, and inform me they have been inoculated with Small-pox virus fix or feven times in the courfe of the laft thirty years, but never could have the difeafe. They had no idea of the reafon why they could not, till Dr. Jenner's firft publication fell in my way, on which I vifited them and obtained the above account. I have fince vaccinated two children and two fervants in this family, who had the difeafe properly, and the gentleman was immediately ftruck with the refemblance of the puftules produced in their arms to thofe which he had feen occafioned by milking difeafed cows.

It would be occupying too much room in your valuable publication to infert more inftances of this kind; or I would relate twenty or thirty, in which perfons who have had the Cow-pox from ten to fifteen years ago, and have been inoculated with Variolous matter, not having any reafon to fuppofe they were fecure by having experienced the other difeafe.

In

* Vide Dr. Pearfon's Pamphlet before alluded to.

In the parifh of Denver, adjoining this place, I inoculated one hundred perfons of all ages; among which I obferved one cafe of fpurious Cow-pox in a maid fervant, and one child in whom I could not fucceed in giving the difeafe; probably from the fyftem being at that time affected with the Chicken-pox.—The young woman was inoculated a fecond time with no better effect; but the mother of the child refufed any farther trial. The remaining 98 perfons went through the difeafe in the ufual way except one little girl, a daughter of Mr. Beetens, who, a week before, had been expofed to variolous infection, the eruption of which came out in confluent numbers on the feventh day after the introduction of Vaccine matter; (which alfo took effect, and the arm met with no interruption in its progrefs from the Variolous difeafe.) Her fifter flept with her the whole time, had an eruption of fome hundred pimples, with widely extended inflammatory margins, but all of them difappeared in three or four days without proceeding to maturation. Query, Does this inftance throw any light upon thofe cafes of eruption fuppofed to have been occafioned by the mixture of the two difeafes, in confequence of a variolated atmofphere? Fearful that the young woman and child abovementioned might take the Small-pox, and be the occafion of unmerited prejudice againft the new inoculation, I took care to apprife the parents of the other ninety-eight children, of the confequences that would follow, if thofe two fhould be expofed to the Variolous infection; at the fame time had no hefitation in pronouncing all the others perfectly fafe from fuch a circumftance. In lefs than three months the maid fervant had the Small-pox in a favourable manner, after attending the funeral of an infant who died of it; and the other child, whom I marked out to them as not fafe, caught the infection and died, whilft the brother, who flept in the fame bed, one of the ninety-eight who had the true Vaccine, remained fecure from the fatal fcourge, as did all the others who had mixed indifcriminately with the moft infectious.

Early in the month of April laft I inoculated twenty-two paupers in the parifh of Shouldham Thorpe; amongft whom was an old woman, aged 80, who was very defirous of having the Small-pox, as fhe had two grand-children in the houfe whofe parents would not confent to have them vaccinated. I had no inclination to indulge her prejudice, knowing the difficulty of managing that clafs of people in refpect to diet and cool treatment; particularly as the diftance from this place would not allow of my feeing her frequently during the eruptive fever. I therefore determined to inoculate *her* with Cow-pock matter, and the *children* with Variolous. The incifions all all took place, and ſhe went through the uſual ſlight indiſpoſition without confinement, while the children were extremely ill four or five days previous to a favourable eruption. As ſoon as I found her arm put on the characteriſtic appearance of Cow-pox, I told her what had been done; when relying on my aſſurance of her ſafety, ſhe has ever ſince expreſſed herſelf quite ſatisfied, as well as the parents of the remaining nineteen children, who were all inoculated with Cow-pock matter, and had the diſeaſe in a proper manner. Many perſons in the ſame village were at that time under Small-pox Inoculation, and were under no reſtraint from mixing with thoſe who were ſuccefsfully vaccinated.

It may be of conſequence to remark, after what length of time perſons expoſed to natural Small-pox may ſafely, and with every hope of intercepting it, be inoculated with Vaccine matter.

In the month of March laſt, ——— Guyton, a girl about 18, having taken the Small-pox in the natural way, was removed from her ſervice, and conveyed to the pariſh of Tottenhill, to her father's houſe; there were ſeven younger children who had never had the diſeaſe. I was deſired to viſit this girl, whoſe diſeaſe was of the confluent kind, on the third day after her removal; and having long wiſhed to aſcertain the liberty that might be taken in theſe inſtances of expoſure to Variolous infection, I inoculated the ſeven children with freſh Vaccine matter, and fortunately ſucceeded in every caſe; and although three * of them did not take place from the firſt inſertion, and were left to be inoculated from thoſe that did, yet they all eſcaped the Small-pox, and went about their uſual employment, which was bird-keeping, or rather ſcaring, and oat-ſetting. In theſe caſes, however, I had matter which was *sure* to ſucceed as far as it would go, or ſhould not have ventured the experiment.

I had never ſeen an inſtance of caſual Cow Pox on the hand of a dairy maid, till about the middle of March laſt; when I was ſent for to Mr. Spink's, a conſiderable farmer at Thorpland, four miles from hence, to viſit his eldeſt child, whom I found with a large ſwelling and painful inflammation ſituated near the eye. Mr. Goddard, a gentleman who lives with me, had ſeen the

* Thoſe who did not take the diſeaſe at firſt were not inoculated till the ſeventh day after; ſo that ten days had elapſed from their firſt expoſure to the Small-pox. There is but one door of entrance to the cottage, and, though ſome care was taken to prevent a general aſſociation of the family, the mother, who nurſed the eldeſt daughter, continued to dreſs and attend upon the younger children.

the child the preceding day, and looked upon the cafe as an accidental inoculation of the Cow Pox; and having had conftant proofs of his judgment and abilities, I went with great pleafure to inveftigate that which I had no doubt had been accurately accounted for by him. The dairy-maid had been defired a week before to apply a plaifter to a flight injury juft above the eye, occafioned by the child falling againft a table. Upon examining her hands, I found feveral large puftules, all of which had been broken; and were now furrounded with an areola to a confiderable extent. The young woman had a week before been much indifpofed, complained of fwelling and pain in the axilla, and there could be no doubt of her having gone through the difeafe in queftion. Upon enquiring how her hands came to be in that ftate, fhe faid it was from milking; and that the cows were many of them very fore with puftules on their paps of the fame appearance. I then procured Mr. Spink's confent to inoculate two younger children, and he as well as Mrs. S——, were much pleafed to fee fimilar puftules produced by my lancet on their arms; but I could not, after feveral attempts, affect the eldeft child, who was ftill fuffering with an angry ulcer from the accidental inoculation. I have every reafon to fuppofe Mr. S—— had the Cow Pox at the fame time with the fervant; he had complained of a ftiffnefs of the arm, and pain from a fmall wound on the infide of the thumb, exactly at the bending of the firft joint; the motion of which prevented the formation of a puftule, or broke it as foon as formed. I have fince inoculated him from the youngeft child, but failed in three or four attempts: he had had an enlargement of the axillary glands, and flight conftitutional derangement. In the fame week, being employed to inoculate a child and two fervants of Mr. Wright's, of Whittington, near Stock, I had an opportunity of feeing another cafe of cafual Cow Pox in the dairy-maid, who had been very ill, and whofe hands exhibited the appearance of genuine Vaccine difeafe about three weeks after the original application of the matter. The cows here were alfo difeafed; but as I never had an opportunity of feeing them, except juft after being milked, the puftules were always broke, and emptied by that operation. Three weeks after this period, fome of the cows ftill being affected, fhe contracted the difeafe again, but the puftules were fomewhat different from the firft cafe, being not fo uniformly circular, more livid, and irregular, and little or no inflammation at their bafes; attended, however, with a very fevere conftitutional illnefs, with ten or fifteen pimples about the fourth day of the fever, which had no characteriftic marks about them; they contained a fmall quantity of fluid at the apex on the fecond day, and maturated on the third. It is to be obferved at this period, the parifh in general was under Small Pox Inoculation, and this young woman, as well as the other fervants and child, were not only frequently going into cottages where the Small Pox then exifted, but they were all, except the child, inoculated by me, from a variolous fubject of their own choofing; which, however, produced no difeafe, and the inoculated parts were fcarcely to be feen at the end of the feventh day. I fhall only mention one more cafe, which I have omitted in its proper place; and which will fhew the fevere trial I have given the Jennerian Difeafe. Being applied to, to inoculate a young woman of the name of Ifaby, in this neighbourhood, and her child, then at the breaft, I employed the ufual means with a vaccinated lancet. The difeafe in the child's arm obferved the ufual progrefs, but the mother's did not take effect.—She, fecretly rejoiced at my failure, faid, "Now, fir, will you give me the Small Pox, and prove that my child is fafe?" I anfwered, I have no objection; but firft, let me make all the children under this roof as fecure as yours, and you fhall be indulged. After three weeks had elapfed, I inoculated this woman with Variolous matter; it produced the ufual fymptoms, under the accuftomed treatment; the eruptive fever was fo confiderable, as to make her repent her choice; but the eruption was diftinct and truly characteriftic; during which the child continued at the breaft, and flept with its mother, having no opportunity of being nurfed elfewhere.

